# Clinical Presentation of Shoulder‐Hand Syndrome: A Systematic Review

**DOI:** 10.1002/ejp.70205

**Published:** 2026-02-04

**Authors:** Rebecca Mountford, Greta Mattocks, Heike L. Rittner, Janne Gierthmühlen, Daniel Ciampi de Andrade, Jee Youn Moon, Tara Packham, Janet H. Bultitude, Michael C. Ferraro, Peter D. Drummond, Arnas Tamasauskas, Andreas Goebel

**Affiliations:** ^1^ Pain Research Institute University of Liverpool Liverpool UK; ^2^ Barts Health NHS Trust London UK; ^3^ Centre for Interdisciplinary Pain Medicine, Dept Anaesthesiology, Intensive Care, Emergency and Pain Medicine University Hospital Wuerzburg Wuerzburg Germany; ^4^ Interdisciplinary Pain Unit, Department of Anesthesiology and Surgical Intensive Care Medicine Universitätsklinikum Schleswig‐Holstein, Campus Kiel Kiel Germany; ^5^ Center for Neuroplasticity and Pain, Health Science and Technology Department, Faculty of Medicine Aalborg University Denmark; ^6^ Department of Anesthesiology and Pain Medicine Seoul National University Hospital Seoul Republic of Korea; ^7^ School of Rehabilitation Sciences, Faculty of Health Sciences McMaster University Hamilton Ontario Canada; ^8^ School of Medicine and Psychology The Australian National University Canberra Australia; ^9^ Department of Psychology University of Bath Bath UK; ^10^ Centre for Pain IMPACT, Neuroscience Research Australia Randwick Australia; ^11^ School of Health Sciences, Faculty of Medicine and Health University of New South Wales Sydney Sydney Australia; ^12^ School of Psychology Murdoch University Perth Australia; ^13^ University of Liverpool Institute of Life Course and Medical Sciences Liverpool UK; ^14^ Walton Centre NHS Foundation Trust Liverpool UK

## Abstract

**Background:**

Shoulder‐hand syndrome (SHS) is a neurological disorder characterised by pain, loss of function, and trophic changes in the shoulder and hand of the affected limb. SHS shares a number of features with Complex Regional Pain Syndrome (CRPS). Historically, several terms have been used interchangeably with SHS, obfuscating clinical presentation. This review aimed to characterise the presentation of SHS to provide a full clinical picture for clinicians and researchers. Furthermore, we aimed to examine whether symptoms differ between triggered and idiopathic SHS, as indicated in previous research.

**Methods:**

A systematic search of three databases (PubMed, Web of Science and Google Scholar) and bibliographies was performed. Articles published from 1940 to 2025 describing symptoms of shoulder‐hand syndrome in any context were screened for eligibility. Papers were excluded if they used alternative terms in place of ‘shoulder‐hand syndrome’, such as reflex sympathetic dystrophy.

**Results:**

16,843 articles were identified, with 33 meeting the inclusion criteria. The clinical presentation of SHS was similar across included studies, with some variations observed between post‐hemiplegic (PH) and post‐myocardial infarction (PM) SHS patients. The predominant symptoms were pain in the shoulder, accompanied by pain and swelling of the hand. PH patients exhibited more trophic symptoms (e.g., nail growth changes, skin thickening), while PM patients demonstrated joint contractures and stiffness.

**Conclusions:**

This review provides a detailed description of the symptoms of shoulder‐hand syndrome, including both triggered and idiopathic cases. We hypothesize that SHS might be a sub‐type of CRPS; however, more research is required to validate this categorization.

**Significance Statement:**

This review provides a detailed description of the symptoms of shoulder‐hand syndrome. This information may be useful for clinicians and researchers examining cases of SHS and possibly contrast this with CRPS. A concerted effort to phenotype these patients, including the influence of inciting events, using modern techniques such as quantitative sensory testing would be useful. We propose that SHS may be a sub‐type of CRPS and if confirmed should be classified accordingly, however more research is needed.

## 1 Introduction

Shoulder‐Hand Syndrome (SHS) is characterised by varying combinations of pain, oedema, trophic abnormalities and loss of function in the shoulder and hand of an affected limb. The term SHS is today sometimes used interchangeably with ‘Complex Regional Pain Syndrome’ for painful symptoms and signs affecting the hand and shoulder of one extremity, particularly when they arise following stroke. While the incidence of CRPS is estimated at 0.07% in the US (Elsharydah et al. 2017), no data are available on the incidence of SHS.

The term SHS emerged sometime in the 1940s. Earliest descriptions of an SHS‐like syndrome were associated with cardiac incidents; Ernestine described shoulder and hand pain post‐myocardial infarction (Ernstene and Kinell 1940). Post‐infarction pain and trophic changes in the hand and shoulder were similarly reported in 1941 (Askey 1941); and the term ‘post‐infarctional sclerodactylia’ was proposed in 1943 (Johnson 1943). Steinbrocker likely coined the term SHS in 1947 (Steinbrocker 1947); in this publication, he observes the similarity of SHS to previously described conditions but notes that these patients are free from signs of cardiac disease. Steinbrocker detailed three stages of SHS in several further publications from 1947 to 1968 (Steinbrocker 1968; Steinbrocker and Argyros 1958; Steinbrocker et al. 1948).

Stage I: Pain and tenderness in the shoulder, swelling of the hand and digits. Sudomotor changes in the affected limb, including hyperhidrosis. Occasional osteoporosis of the shoulder seen on X‐rays.

Stage II: Symptoms either progress or spontaneously resolve, particularly in the hand. If symptoms progress, pain in the shoulder worsens, and the pain and swelling of the hand become more advanced, accompanied by joint contractures. The skin of the affected limb is shiny; hair and nail growth are altered.

Stage III: In stage III, severe pain in the shoulder and hand is uncommon. However, muscle dystrophy and digital contractures are now permanent, meaning the affected limb is irreversibly disabled. The occasional osteoporosis that was seen on X‐rays during stage I and II is significantly more marked, giving a “ground‐glass” appearance to the humeral head and fingers.

To our knowledge, no systematic review examining the clinical presentation of SHS is available. One previous review examined the pathophysiology and treatment of SHS (Geurts et al. 2000). Other similar systematic reviews have focused on treatment options such as acupuncture (Peng et al. 2017) or Chinese medicine (Rui et al. 2020). Additional reviews on SHS and Chinese medicine were identified but were not available in English.

As the main objective of this review was to consolidate all available information on SHS to provide a complete picture of the clinical presentation of this rare condition, the secondary objective was to assess potential differences between idiopathic and triggered SHS, particularly cases following stroke or myocardial infarction. Finally, we aimed to contextualise the symptoms of SHS within CRPS and explore if these conditions are related; for example, with SHS potentially being a sub‐type of CRPS.

## 2 Methods

This review was conducted and reported in accordance with the Preferred Reporting Items for Systematic Reviews and Meta‐analyses (PRISMA) guidelines (Page et al. 2021). The protocol was registered on PROSPERO (ID: CRD42024542229).

### 2.1 Literature Search Strategy

An electronic database search was conducted across PubMed, Web of Science, and Google Scholar for articles published between 1940 and 2025. Bibliographies of articles that were identified in the database search were also screened for relevant articles. The final search was performed in June 2025. Keywords and phrases used across these three databases included ‘shoulder hand syndrome,’ ‘shoulder‐hand syndrome,’ ‘shoulder hand syndrome hemiplegic or hemiplegia,’ ‘shoulder hand syndrome stroke,’ and ‘shoulder hand syndrome myocardial or myocardial infarction.’ Throughout the search, keywords were separated by Boolean phrases (“AND,” “OR,” “NOT”). The word “impingement” (i.e., shoulder impingement syndrome) was excluded from all keyword searches, as preliminary database screening identified that Google Scholar did not distinguish between shoulder impingement and shoulder‐hand syndrome. Google Scholar was selected as one of the databases to identify possible grey literature (Paez 2017). Searches were conducted by a single researcher (RM).

### 2.2 Inclusion and Exclusion Criteria

Articles were included if they were published in English and involved or described adult human participants. Articles were included if they described direct contact with participants or participant data (e.g., case studies, retrospective studies); reviews, systematic reviews, and meta‐analyses were excluded. All included studies had to use the term ‘shoulder‐hand syndrome’ within the title or text. Papers using exclusively alternate terms such as RSD, CRPS, frozen shoulder, or shoulder impingement syndrome were excluded. Articles that described other conditions *alongside* SHS or referred to a combination of syndromes (such as CRPS‐SHS) were included as long as the SHS patients were described individually, and the criteria for SHS were clearly defined. Articles had to detail the clinical presentation of SHS investigated by either referencing previously published descriptions of SHS symptoms as inclusion criteria (e.g., by Steinbrocker) or describing any idiosyncratic SHS criteria used.

**FIGURE 1:**
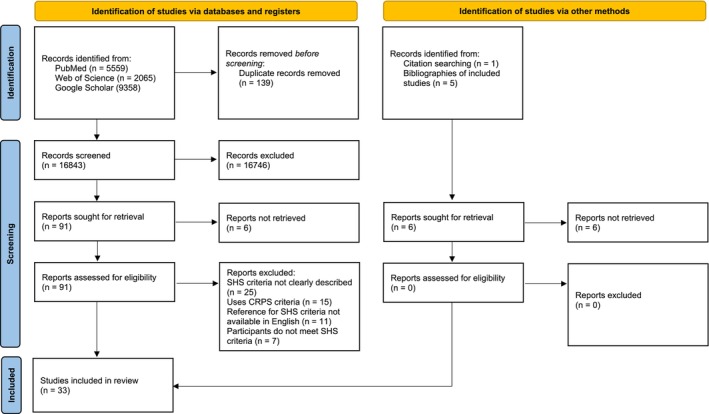
PRISMA Flowchart of study screening and selection.

All articles identified through database or reference list searches were retrieved and screened by one reviewer (RM). Titles and abstracts were assessed first, then full texts were obtained if the study appeared to meet the inclusion criteria. Two reviewers (RM and GM) independently screened the full texts for eligibility with disagreements resolved by discussion. The screening process is represented in Figure 1 with a PRISMA flow diagram.

### 2.3 Data Collection

One reviewer (RM) collected and extracted data to an Excel spreadsheet. Extracted data included: year of publication, geographical location of the study, number of participants, mean age and age range of participants, sex of participants, male/female ratio of participants, study design, diagnostic criteria for SHS used by the researchers, and SHS symptoms listed by affected region of the limb (shoulder, elbow, wrist, hand, or non‐specific if the study only referred to the ‘affected limb’). A second reviewer (GM) screened this spreadsheet to ensure all the data was accurate. No additional data was requested from the study investigators of any of the included papers. Articles with missing or incomplete information on the criteria used to identify participants with shoulder‐hand syndrome were excluded according to the eligibility criteria.

## 3 Results

### 3.1 Search Results

A total of 16,982 articles were identified in the search across three databases (PubMed (*n* = 5559), Web of Science (*n* = 2065), and Google Scholar (*n* = 9358)). Screening of titles and abstracts, after the removal of duplicates, resulted in the exclusion of 16,746 papers, leaving ninety‐seven potentially eligible articles. After a full‐text review of these manuscripts, thirty‐three were included in this systematic review. Six articles were identified in this search that we were not able to access. The author team enlisted the help of the University of Liverpool library but were unable to access the full texts due to the lack of information on the authors. The titles and publication dates of these six studies can be found in Table S6.

This review was descriptive in nature and did not involve an assessment of study quality.

### 3.2 Study Characteristics

The thirty‐three included studies were published between 1947 and 2020. They consisted of eight case reports (studies were categorised as case reports if they reported on a single participant) (Akasbi et al. 2013; Kammerling et al. 1950; Low et al. 1978; Massarotti et al. 2008; McGill and Calman 1985; Reddy 1985; Santamato et al. 2009; Valley et al. 1994), twelve case series (Amick et al. 1966; Baer 1966; Cohen et al. 1960; Edeiken 1957; Mowat 1974; Rosen and Graham 1957; Russek et al. 1953; Swan and McGowan 1951; Taggart et al. 1984; Taylor 1958; Thompson 1961; Walker et al. 1983), two case–control studies (Aisen et al. 1995; Hesse et al. 1995), three cross‐sectional studies (Aisen and Aisen 1994; Chalsen et al. 1987; van der Korst et al. 1966), four non‐randomised clinical trials (Braus et al. 1994; De Santis et al. 2000; Dekker et al. 1997; Pan et al. 2020), two controlled before‐and‐after studies (non‐randomised) (Kondo et al. 2001; Wang et al. 2015), one retrospective study (Davis et al. 1977) and one cluster‐randomised clinical trial (Hartwig et al. 2012). Study characteristics for each included article are listed below in Table 1. In the absence of agreed diagnostic criteria, the SHS criteria used by each study are listed in Appendix S1.

**TABLE 1:** Study characteristics for all included articles.

First author	Year	Region	Study design	Sample size	Age		Sex		Associated condition
					Mean	Range	Male	Female	
Kammerling, E.	1950	USA	Case report	1	64	—	1	—	Myocardial infarction
Swan, D.	1951	USA	Case series	3	60	56–63	2	1	Myocardial infarction
Russek, H.	1953	USA	Case series	17	66.4	48–62	—	—	Myocardial infarction
Edeiken, J.	1957	USA	Case series	42	—	—	—	—	Myocardial infarction
Rosen, P.	1957	Canada	Case series	73	63	31–80	—	—	Several^a^
Taylor, J.	1958	UK	Case series	6	—	—	—	—	Several^b^
Cohen, A.	1960	USA	Case series	12	62.3^g^	50–78^g^	2^g^	1^g^	Several^c^
Thompson, R.	1961	UK	Case series	17	—	18–80	9	8	Several^d^
Amick, L.	1966	USA	Case series	21	46^g^	13–67^g^	2^g^	1^g^	Psychological
Baer, R.	1966	USA	Case series	3	60.3^g^	58–62^g^	2^g^	1^g^	Myocardial, injury, idiopathic
Van der Korst, J.	1966	Netherlands	Cross‐sectional	75	58.5	22–81	41	34	Several^e^
Mowat, A.	1974	UK	Case series	3	55.6	51–65	—	2	Several^f^
Davis, S.	1977	USA	Retrospective	540	—	50–79^g^	43^g^	25^g^	Stroke
Low, L.	1978	USA	Case report	1	27	—	0	1	Sterilisation
Walker, J.	1983	USA	Case series	3	53.6	51–56	1	2	Cerebral neoplasms
Taggart, A.	1984	UK	Case series	2	50	42–58	0	2	Ovarian carcinoma
McGill, P.	1985	UK	Case report	1	43	—	0	1	Ovarian carcinoma
Reddy, M.	1985	USA	Case report	1	53	—	0	1	Phenobarbital
Chalsen, G.	1987	USA	Cross‐sectional	41	65.5	—	19	22	Stroke
Aisen, P.	1994	USA	Cross‐sectional	43	39	16–86	32	11	Spinal cord injury
Braus, D.	1994	Germany	NR clinical trial	132	62.4	—	90	42	Stroke
Valley, M.	1994	USA	Case report	1	35	—	0	1	Brachial plexus injury
Aisen, M.	1995	USA	Case–control	14	47.8	17–70	11	3	Spinal cord injury
Hesse, S.	1995	Germany	Case–control	39	60.5	43–79	26	13	Stroke
Dekker, J.	1997	Netherlands	NR clinical trial	9	—	—	—	—	Stroke
Desantis, A.	2000	Italy	NR clinical trial	234	47.5	2–88	155	119	Phenobarbital
Kondo, I.	2001	Japan	Before & after	152	62.3	—	62	90	Stroke
Massarotti, M.	2008	Italy	Case report	1	67	—	0	1	Gastric cancer
Santamato, A.	2009	Italy	Case report	1	68	—	0	1	Stroke
Akasbi, N.	2010	Morocco	Case report	1	70	—	0	1	Lung cancer
Hartwig, M.	2012	Germany	R clinical trial	41	64.5	—	23	18	Stroke
Wang, J.	2015	China	Before & after	120	61	—	65	55	Stroke
Pan, J.	2020	China	NR clinical trial	90	61.2	48–75	57	33	Stroke

^a^ Rosen, P. 1957: Conditions associated with the development of shoulder‐hand syndrome were categorised under five subheadings:
Idiopathic (*n* = 10). Peripheral lesions (trauma (*n* = 5), cervical disc degeneration (*n* = 10), tuberculoma (*n* = 10), recent myocardial infarction (within eight weeks, *n* = 18), former myocardial infarction (up to two years previously, *n* = 3), hypertension (*n* = 3), pulmonary fibrosis (*n* = 1) and signs of heart disease (*n* = 6)). Lesions of the spinal cord – Quadriplegia (*n* = 4). Cerebral lesions (cerebral tumour (*n* = 1), stroke (*n* = 4), subarachnoid haemorrhage (*n* = 3), head injury (*n* = 1)). Miscellaneous (prolonged best rest ≥ 6 months, also includes cirrhosis and diverticulitis (*n* = 2), abdominal surgery (*n* = 1), block dissection of neck (*n* = 1) and fractured neck (*n* = 1)).

^b^ Taylor, J. 1958: 4/6 cases were associated with physical trauma, one case linked to myocardial infarction, and one case was deemed idiopathic.

^c^ Cohen, A. 1960: 6/12 idiopathic, three cases attributed to myocardial infarction, the final three cases were linked to a stroke.

^d^ Thompson, R. 1961: Epilepsy (*n* = 6), meningoencephalitis (*n* = 1), myocardial infarction (*n* = 2), cervical spondylosis (*n* = 1), herpes zoster (*n* = 2), ‘drug coma and trauma’ (*n* = 1), stroke (*n* = 1), epithelioma (*n* = 1), idiopathic (*n* = 1), uncertain; possible link to coronary artery disease and/or epilepsy (*n* = 1).

^e^ Van der Korst, J. 1966: Several drugs were associated with the development of shoulder‐hand syndrome; phenobarbital (*n* = 19), ‘unspecified sedatives’ (*n* = 5), phenytoin (*n* = 4), and isoniazid (*n* = 4).

^f^ Mowat, A. 1974: 1/3 idiopathic, 1/3 stroke, 1/3 phenobarbital prescribed for epilepsy.

^g^ Indicates data was not available for all cases.

Most *case reports* described SHS developing alongside, or possibly as a result of, different conditions. SHS in conjunction with a form of cancer was reported in three case reports: ovarian carcinoma (*n* = 1) (McGill and Calman 1985), gastric cancer (*n* = 1) (Massarotti et al. 2008), and lung cancer (*n* = 1) (Akasbi et al. 2013). SHS was also associated with myocardial infarction (*n* = 1) (Kammerling et al. 1950), stroke (*n* = 1) (Santamato et al. 2009), brachial plexus injury (*n* = 1) (Valley et al. 1994), and laparoscopic sterilisation (*n* = 1) (Low et al. 1978). One case report described SHS developing after a patient was prescribed phenobarbital as a treatment for epilepsy (*n* = 1) (Reddy 1985). Two *case series* discussed SHS associated with cancer, one with ovarian carcinoma (*n* = 2) (Taggart et al. 1984), and the other intracranial neoplasms (*n* = 3) (Walker et al. 1983); several case series attributed SHS, at least in part, to a previous myocardial infarction (combined *n* = 89) (Cohen et al. 1960; Edeiken 1957; Rosen and Graham 1957; Russek et al. 1953; Swan and McGowan 1951; Taylor 1958; Thompson 1961). The remaining articles listed a range of associated and possibly causative conditions, such as trauma, cirrhosis, and epilepsy (combined *n* = 132) (Amick et al. 1966; Baer 1966; Cohen et al. 1960; Mowat 1974; Rosen and Graham 1957; Taylor 1958; Thompson 1961).

The first *case–control study* observed the different sympathetic skin responses of patients with a cervical spinal cord injury who developed SHS compared to those who didn't and included additional results of re‐testing patients after they underwent treatment with prednisone (patients *n* = 7/controls *n* = 7) (Aisen et al. 1995); the second case–control study similarly examined sympathetic skin response, but in hemiplegic SHS patients (patients *n* = 21/controls *n* = 18) (Hesse et al. 1995). One *cross‐sectional study* investigated SHS in cervical spinal cord injury (*n* = 43) (Aisen and Aisen 1994), one examined the correlation between phenobarbital for the treatment of epilepsy and SHS development (*n* = 75) (van der Korst et al. 1966), and another reported SHS prevalence in a stroke rehabilitation centre (*n* = 41) (Chalsen et al. 1987). Two *clinical trials* described the effect of different pharmaceutical treatments of SHS symptoms in post‐hemiplegic patients, one using oral corticosteroids (*n* = 132) (Braus et al. 1994), and another intra‐articular corticosteroid injection (*n* = 9) (Dekker et al. 1997); a third clinical trial assessed the link between barbiturates and development of SHS in neurosurgical patients (defined by the authors as patients with head injuries, intracranial meningiomas, and intracranial ruptured aneurysms) (*n* = 234) (De Santis et al. 2000). The final clinical trial compared traditional Chinese medicine (moxibustion) and acupuncture separately and combined in post‐hemiplegic SHS patients (*n* = 90) (Pan et al. 2020). Both *before‐and‐after studies* described the association between SHS and stroke. One focused on the prevention of SHS (*n* = 152) (Kondo et al. 2001), the other applied superficial needling to their patient cohort (*n* = 120) (Wang et al. 2015). Similarly, the 1997 *retrospective study* (*n* = 540) assessed the incidence of SHS in hemiplegic patients over a five‐year period (Davis et al. 1977). The *randomised clinical trial* investigated the effectiveness of an external shoulder joint support device on preventing partial shoulder joint dislocation in post‐hemiplegic SHS patients (*n* = 41) (Hartwig et al. 2012).

### 3.3 Reported Symptoms of SHS

The most frequently reported symptoms across all included articles were shoulder pain (97%), swelling in the hand (82%), hand pain (79%), limited ROM in the shoulder (79%), and shoulder tenderness (42%). No data were available outlining whether shoulder and hand pain occurred concomitantly. The symptoms reported by each included article are described in Table 2, 3, 4. Of the thirty‐three included studies, fifteen reported X‐ray results. Ten articles observed osteoporosis in the shoulder, one reported osteopenia in the shoulder, and five reported osteoporosis of the hand. No articles provided information on X‐ray results for both the shoulder and hand. Two articles reported ‘demineralization’ of the arm and shoulder (*n* = 1), or demineralization of the wrists and hands (*n* = 1). Interestingly, some reports separately described digit symptoms and hand symptoms (unlike as would be reported for people with CRPS); therefore, we have divided hand and digits into separate tables below.

**TABLE 2:** Shoulder symptoms reported across all included articles.

1st Author	Pain (%)	Limited ROM (%)	Stiffness (%)	Tenderness (%)	Pain and limited ROM associated with specific movement			Osteoporosis on X‐ray (%)	Osteopenia on X‐ray (%)
					Humeral abduction (%)	Flexion rotation (%)	External rotation (%)		
Kammerling, E.**	100	—	—	—	—	—	—	—	—
Swan, D.**	100	100	—	100	—	—	—	—	—
Russek, H.	100	100	—	100	—	—	—	100	—
Edeiken, J.	100	100	—	100	—	—	—	—	—
Rosen, P.	100	100	—	—	100	100	—	—	—
Taylor, J.*	100	100	—	100	—	—	—	—	—
Cohen, A.	100	100	—	100	—	—	—	—	—
Thompson, R.	100	100	—	100	—	—	—	—	—
Amick, L.	100	100	—	50	—	—	—	50	—
Baer, R.**	100	100	—	100	100	100	100	—	—
Van der Korst, J.	100	—	100	—	—	—	—	—	—
Mowat, A.**	100	100	33	—	—	—	—	—	—
Davis, S.	100	100	—	—	100	100	100	100	—
Low, L.**	100	100	—	—	—	—	—	100	—
Walker, J.**	100	100	66	100	—	33	33	0	—
Taggart, A.**	—	100	50	—	—	—	—	—	—
McGill, P.**	100	—	100	—	—	—	—	—	—
Reddy, M.**	100	100	100	—	—	100	100	100	—
Chalsen, G.	100	100	—	100	—	—	—	100	—
Aisen, P.	88	—	—	—	—	—	—	13/19	—
Braus, D.	100	100	—	100	100	100	100	—	—
Valley, M.**	100	100	—	100	—	—	—	—	—
Aisen, M.	100	—	—	—	—	—	—	100	—
Hesse, S.	100	—	—	100	100	100	100	—	—
Dekker, J.*	100	100	—	—	—	—	—	0	—
Desantis, A.	100	100	—	100	—	—	—	—	—
Kondo, I.	100	—	—	—	—	—	—	—	—
Massarotti, M.**	100	100	—	—	—	—	—	100	—
Santamato, A.**	100	100	100	—	—	—	—	—	—
Akasabi, N.**	100	100	—	—	—	—	—	—	100
Hartwig, M.	100	100	—	—	—	—	—	—	—
Wang, J.	100	100	—	—	—	—	—	—	—
Pan, J.	50	50	—	—	—	—	—	—	—

*Note:* *n = ≤ 10, **n = ≤ 3.

**TABLE 3:** Hand symptoms reported across all included articles.

1st Author	Pain (%)	Swelling (%)	Stiffness (%)	Tenderness (%)	Limited ROM (%)	Tissue atrophy (%)	Flexion contractures/deformities (%)	Discolouration (%)	Temp. changes (%)	Sweating increase/reduction (%)	Shiny skin (%)	Skin thickening (%)	Non‐specific trophic changes (%)	Osteoporosis on X‐ray (%)
Kammerling, E.**	100	100	—	100	—	100	—	100	—	—	—	—	—	100
Swan, D.**	100	100	33	—	33	—	—	33	100	—	—	—	100	—
Russek, H.	100	100	100	—	100	—	100	100	100	—	100	—	100	—
Edeiken, J.	100	100	100	—	—	—	12	—	—	—	—	—	—	—
Rosen, P.	94	100	—	—	—	—	—	100	—	—	—	100	—	24/39
Taylor, J.*	100	100	—	—	—	—	—	—	—	—	—	—	—
Cohen, A.	100	100	—	—	33	—	—	—	—	—	—	—	—	—
Thompson, R.	—	100	—	100	—	—	—	100	—	—	—	—	—	—
Amick, L.	—	50	—	50	25	50	50	—	25	25	25	—	25	—
Baer, R.**	100	—	66	—	66	—	100	33	—	66	—	100	—	—
Van der Korst, J.	—	100	—	—	—	100	—	—	—	—	—	—	—	—
Mowat, A.**	33	100	100	33	66	—	33	66	66	100	66	—	—	33
Davis, S.	—	100	—	—	—	—	—	—	—	—	—	—	—	—
Low, L.**	100	100	—	—	100	—	—	—	100	100	—	—	—	—
Walker, J.**	100	100	—	—	33	—	33	33	—	—	—	—	—	0
Taggart, A.**	50	100	—	—	—	50	50	50	—	—	50	100	—	—
McGill, P.**	100	100	—	—	—	100	100	—	—	—	—	—	—	—
Reddy, M.**	100	100	100	—	—	—	—	—	—	—	—	—	—	—
Chalsen, G.	100	100	—	100	—	—	—	100	100	—	—	—	100	—
Aisen, P.	49	49	—	—	—	—	—	—	44	44	—	—	14	—
Braus, D.	100	100	—	—	—	—	—	100	100	—	—	—	—	—
Valley, M.**	100	—	—	—	—	—	—	—	100	—	—	—	—	—
Aisen, M.	100	100	—	—	—	—	—	—	—	—	—	—	100	—
Hesse, S.	100	100	—	—	—	—	—	100	100	100	—	—	—	—
Dekker, J.*	100	100	—	—	—	—	—	100	—	—	—	—	—	—
Desantis, A.	100	—	—	—	100	—	—	—	100	—	—	—	100	—
Kondo, I.	100	—	—	—	—	—	—	100	—	—	—	—	—	—
Massarotti, M.**	100	100	—	—	—	—	—	—	—	—	—	—	—	—
Santamato, A.**	100	—	—	—	—	—	—	—	—	100	—	—	100	100
Akasabi, N.**	—	100	—	—	100	—	—	—	—	100	—	—	—	—
Hartwig, M.	100	100	—	—	100	—	—	—	—	—	—	—	100	—
Wang, J.	—	100	—	—	—	—	—	100	100	100	—	100	—	100
Pan, J.	—	—	50	—	—	—	—	—	—	—	—	—	—	—

*Note:* *n = ≤ 10, **n = ≤ 3.

**TABLE 4:** Digit symptoms reported across all included articles.

1st Author	Pain (%)	Swelling (%)	Stiffness (%)	Limited ROM (%)	Flexion contracture/deformities (%)	Discolouration (%)	Skin thickening (%)	Shiny skin (%)	Nail growth changes (%)
Kammerling, E.**	—	—	—	—	—	—	—	—	—
Swan, D.**	100	66	100	66	33	33	—	—	—
Russek, H.	100	100	100	100	100	—	—	—	—
Edeiken, J.	100	—	100	—	—	—	—	—	—
Rosen, P.	—	—	—	100	—	—	—	—	—
Taylor, J.*	100	—	—	100	—	—	—	—	—
Cohen, A.	—	—	—	33	—	—	—	—	—
Thompson, R.	—	100	—	—	—	100	—	—	—
Amick, L.	—	—	—	—	—	—	—	—	—
Baer, R.**	33	—	—	66	66	—	—	66	—
Van der Korst, J.	—	—	—	—	—	—	—	—	—
Mowat, A.**	—	33	—	—	33	—	33	—	—
Davis, S.	100	100	—	100	—	—	100	—	—
Low, L.**	100	—	—	100	—	—	—	—	—
Walker, J.**	—	—	—	—	66	—	—	—	—
Taggart, A.**	—	—	50	—	50	—	—	—	—
McGill, P.**	—	—	—	—	—	—	—	—	—
Reddy, M.**	—	100	—	100	100	—	—	—	—
Chalsen, G.	100	100	—	—	100	—	—	—	—
Aisen, P.	—	—	—	—	—	—	—	—	—
Braus, D.	—	100	—	—	—	—	100	—	100
Valley, M.**	—	—	—	—	—	—	—	—	—
Aisen, M.	—	—	—	—	—	—	—	—	75
Hesse, S.	—	100	—	—	—	—	50	—	50
Dekker, J.*	—	100	—	—	—	—	—	—	—
Desantis, A.	—	—	—	—	—	—	—	—	—
Kondo, I.	100	100	—	—	—	100	—	—	—
Massarotti, M.**	—	—	—	—	100	—	—	—	—
Santamato, A.**	—	—	—	—	100	—	—	—	100
Akasabi, N.**	—	—	—	—	—	—	—	—	—
Hartwig, M.	—	100	—	—	—	—	—	—	—
Wang, J.	—	100	—	—	—	—	100	—	100
Pan, J.	—	—	—	50	100	50	50	—	—

*Note:* *n = ≤ 10, **n = ≤ 3.

Information on the time course of SHS development was incomplete. Time intervals between the inciting incident (e.g., myocardial infarction) and development of SHS symptoms ranged from a few weeks to several months, and such developments followed a diverse range of medical and rehabilitative treatments. Many articles did not provide any follow‐up or only described vague timelines of symptom development. Outcomes that were reported varied widely from complete resolution of symptoms to long‐lasting hand/digit contractures. Further information on the time course of SHS in the included articles is available in Appendix S3.

### 3.4 SHS Associated With Stroke and Myocardial Infarction

The reported symptoms of SHS varied slightly between the two most common trigger events of stroke (post‐stroke/hemiplegic, PH, 10 publications) and myocardial infarction (post‐myocardial infarction, PM, 5 articles). Figure 1 display symptoms reported for each trigger, categorised by body region affected.

Three of the ten included articles describing SHS after stroke (Kondo et al. 2001; Pan et al. 2020; Santamato et al. 2009) describe *hemiplegic* patients (total or severe unilateral paralysis after stroke), whereas three articles (Dekker et al. 1997; Hartwig et al. 2012; Hesse et al. 1995) describe *hemiparetic* (unilateral partial paralysis or weakness after stroke). Davis reported 29.5% hemiplegic and 70.5% hemiparetic SHS patients (Davis et al. 1977). The remaining three papers (Braus et al. 1994; Chalsen et al. 1987; Wang et al. 2015) do not specify the hemiplegic or hemiparetic nature of motor defects. As there is inconsistent use of these terms, we use ‘post‐hemiplegic’ (PH) throughout.

We found differences between reported signs and symptoms in the PH and PM studies, the most prominent being that hand and finger stiffness was only frequently reported in PM studies. Given the association between myocardial infarction and shoulder pain, we investigated laterality in the relevant studies. Swan and McGowan (1951) reported 1/3 of cases were contralateral, 2/3 ipsilateral; Edeiken (1957) stated “they found no correlation.” No other included studies make mention of it.

## 4 CRPS After Stroke

We found that the use of the term SHS is uncommon in English language publications post‐2000 whereas ‘CRPS after stroke’ or related terms have become more common. The term CRPS was first coined in 1994 (Harden et al. 2010). We therefore conducted a post hoc PubMed search to quantify the number of reported studies in such publications using the terms *post‐stroke complex regional pain syndrome/CRPS*, or ‘complex regional pain syndrome/CRPS after stroke’ between 2000 and 2025, and to assess the reported patient phenotypes. Further information is available in Appendix S2. We identified 28 relevant articles, in the majority from groups in Korea, Turkey and India; of these studies, only nine report shoulder symptoms, predominantly shoulder pain and limited range of motion of the shoulder, and four of these papers incorporate the term Shoulder‐hands syndrome.

## 5 Discussion

**FIGURE 2:**
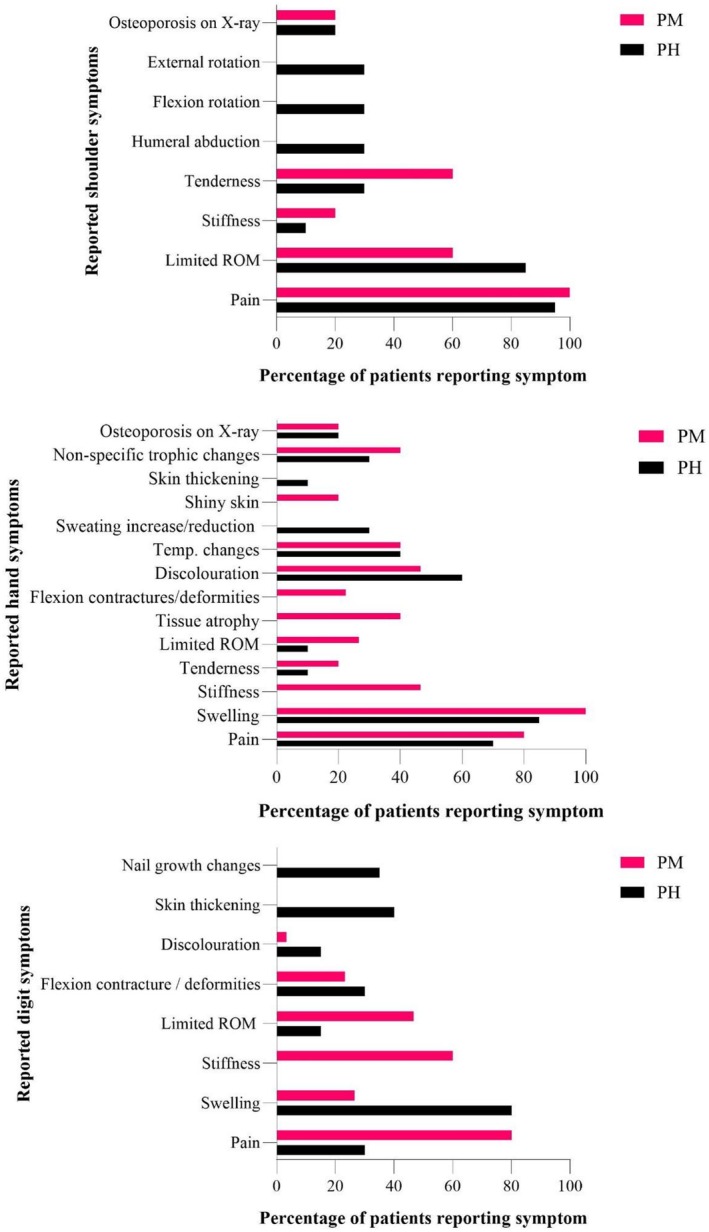
Reported *shoulder*, *hand* and *digit* symptoms for post‐hemiplegic and post‐myocardial infarction SHS patients.

This systematic review aimed to characterise the clinical presentation of shoulder‐hand syndrome, determine whether SHS features differed if associated with the two most commonly reported triggers, stroke and myocardial infarction, and put these presentations into context with typical presentations of CRPS outlined in the Budapest criteria. The 32 included articles, published between 1947 and 2024, chiefly describe symptoms that mirror Steinbrocker's early criteria (Steinbrocker 1947), with limited differences observed between SHS associated with stroke and myocardial infarction (Figure 2). These symptoms were not present in those with post‐myocardial infarction SHS. PM The clinical presentation of SHS appeared consistent across triggering/co‐morbid scenarios such as cancer, cervical spinal cord injury, use of certain medications and rare conditions, but this was informed by very limited data. Of the five articles that reported idiopathic cases of SHS (combined *n* = 19 patients), symptoms were practically identical to the post‐trigger cases; this is detailed in Tables 2, 3, 4.

The Budapest criteria for CRPS cover all major hand symptoms denoted for SHS in Steinbrocker's original criteria; vasomotor, sudomotor, sensory, and motor disturbances (Harden et al. 2022); however, they do not require the presence of shoulder symptoms or osteoporosis or osteopenia in the affected limb, although the latter is frequently observed (Abe et al. 2011; Bazika‐Gerasch et al. 2019; Goh et al. 2017; Moriwaki et al. 1997; Mussawy et al. 2017). Differences between SHS and CRPS criteria extend to the concept of disease progression. The three stages described by Steinbrocker originally in 1947 (Steinbrocker 1947) appear to also apply to the subsequently described SHS cases. In contrast, modern understanding of CRPS does not involve formal stages, though CRPS, unlike SHS, is sometimes considered to have subgroups such as early and persistent, or warm and cold, without obligatory sequence between them (Goebel et al. 2021; Taylor et al. 2021). Of note, while earlier ‘IASP’ and ‘Veldman’ diagnostic criteria have been applied to CRPS after stroke (Pertoldi and Di Benedetto 2006), there is limited validation of the Budapest criteria for this population (Cacchio et al. 2009; Oh et al. 2019; Pervane Vural et al. 2016).

The role of physical trauma appears to differ between CRPS and SHS. Whereas triggering physical limb trauma is typical in CRPS preceding 90% of cases (Veldman et al. 1993), such trauma is not part of Steinbrocker criteria and we did not find physical trigger‐trauma noted in any of the reviewed publications. Braus’ 1994 report, however, sets this situation potentially into a different context (Braus et al. 1994). The incidence of SHS in their cohort of post‐stroke patients declined dramatically with limb‐protective measures taken to prevent small trauma to the affected and, due to its paralysis, less protected limb. They postulate that such trauma occurs to ‘the limb’, rather than the shoulder specifically, and they further suggest that stroke, through the inability to move the stroke‐affected limb, predisposes to the experience of small limb trauma; they consequently speculate that the trigger for SHS development post stroke is not stroke but physical trauma facilitated by stroke.

Another uncertainty concerns Steinbrocker's view that the shoulder pain in SHS is a *primary feature of the condition*. That is, shoulder pain is not secondary to (i) muscle contractions caused by an intensely painful hand condition as often observed in CRPS, or (ii) independent stroke/MI‐induced anatomical shoulder changes (Albazaz et al. 2008), or (iii) seemingly independent pathology such as tendinitis (Veldman and Goris 1995).

The nature of SHS pain might be clarified with modern pain assessment methods. For example, shoulder pain quality and QST profiles might be compared between SHS cases following stroke/MI and these alternative putative causes. Comparison of phenotypic characteristics between right and left SHS after MI would also appear pertinent—might shoulder pain in some of these cases be referred from damaged or inflamed tissue in the heart to the shoulder? Investigation into the role of disuse appears pertinent for both stroke and MI‐related SHS; however, the observed reduction of cases after protection from limb‐trauma by Braus et al. (1994), and the historical accounts of idiopathic SHS (Cohen et al. 1960; Mowat 1974; Rosen and Graham 1957; Taylor 1958; Thompson 1961) would suggest that not all cases will be secondary solely to disuse. A related question is whether there are commonalities between the shoulder aspect of SHS and ‘frozen shoulder’, another poorly understood, highly painful, exercise‐responsive shoulder condition (Mertens et al. 2022; Millar et al. 2022).

A significant finding of this review was the lack of *recent* literature concerning the clinical presentation of shoulder‐hand syndrome. This presents a significant gap in available resources and severely limits our understanding of the presentations which these earlier authors have seen and reported. We found that those more recent reports that use terms like ‘CRPS after stroke’ only inconsistently report shoulder problems, which are of course also not part of the Budapest criteria.

The available SHS research reports had been conducted with a wide range of aims, and this, together with differing professional backgrounds of investigators (such as neurologists or cardiologists) may have influenced details of symptom description. Some symptoms may have been omitted if not deemed relevant, or others may have been grouped together; for example, several articles have detailed hand and digital symptoms separately, whilst others only reported hand symptoms. It is likely that the latter bundled hand and digital symptoms together.

Of the 32 included articles, only seven were published post‐2000. In contrast, prompted by observation of articles only published in Chinese, a brief search of the China National Knowledge Infrastructure reveals nearly 3000 SHS papers published since 2000 (including systematic reviews, such as (Peng et al. 2017; Shi et al. 2025; Shi et al. 2023)). Article titles are often available in English; however, where, more typically, full manuscripts were exclusively printed in Chinese their content was inaccessible to us. While there is currently no available data on the prevalence of SHS in Europe and North America, the prevalence of SHS is estimated between 12.5% and 74.1% post‐stroke in China (Yu et al. 2023). It is interesting to note that the term SHS is, therefore, used much more commonly in Chinese literature compared to English. Although a 2013 paper indicated that Chinese populations may have a higher incidence of stroke when compared to white European communities (33% and 12%, respectively), this difference is unlikely to account for this dramatic publication disparity (Tsai et al. 2013); differences in rehabilitative interventions might be speculated to contribute to this phenomenon. The criteria used for diagnosing SHS syndrome in these Chinese articles includes the Budapest criteria for CRPS (Shi et al. 2023; Yu et al. 2023) and Chinese Rehabilitation Research Centre for SHS (criteria not accessible in English) (Li et al. 2024). The existence of a Research Centre for SHS within China may further indicate that SHS incidence is greater in China.

Future shoulder‐hand syndrome research should be focused on describing the clinical phenomenology of patients presenting with painful hands by actively including both enquiry of shoulder symptoms and assessment of shoulder signs despite this not being needed for Budapest criteria; this may be particularly pertinent following stroke or myocardial infarction but where possible also following somatic trauma; some of us are aware of such symptoms occurring with some regularity in this way. Such research should also aim to deliver longitudinal data (in how much does this condition resolve, and how fast) and ideally should investigate affected hands and shoulders with quantitative sensory testing—such data can then be compared with typical CRPS profiles (Husk et al. 2025). Currently, it is of course also unclear whether SHS should be managed similar independent of trigger, and whether management would need to differ from CRPS. Should SHS patients be included in CRPS research? Further research is required to answer these questions.

## 6 Limitations

This review had several limitations. Only three databases were used in the literature search; consequently, not all available evidence may have been captured in this search. Additionally, a single researcher was responsible for abstract and title screening, meaning some eligible articles may have been missed or erroneously excluded (Mahtani et al. 2020). Papers using the term post‐stroke CRPS were reviewed post hoc; therefore, our comparisons (appendix) may be biased. Additionally, reports using terms other than SHS or CRPS when describing a shoulder‐hand phenomenon would have been missed. As the majority of included articles were published between 1947 and 1997, population changes and changes in medical practice over the last 70+ years (including modern rehabilitation approaches after MI and stroke) may limit the relevance of these studies in modern populations. Because the common use of the term SHS in China was an unexpected finding, we did not investigate this phenomenon in more detail.

## 7 Conclusions

This review provides a detailed description of the clinical phenomenon of symptoms of shoulder‐hand syndrome. This information may be useful for clinicians and researchers examining cases of SHS and possibly wishing to understand the condition in the context of CRPS. A concerted effort to phenotype these patients, including the influence of inciting events, using modern techniques such as quantitative sensory testing would be useful. We propose that SHS may be a sub‐type of CRPS and, if confirmed, should be classified accordingly; however, more research is needed.

## Author Contributions

The research question for this review was conceptualised by Andreas Goebel. Literature search and data extraction were performed by Rebecca Mountford and Greta Mattocks. Data analysis was performed by Rebecca Mountford and Arnas Tamasauskas. Andreas Goebel and Rebecca Mountford prepared the original draft of this manuscript, which was edited by Heike L. Rittner, Janne Gierthmühlen, Daniel Ciampi de Andrade, Jee Youn Moon, Tara Packham, Janet H. Bultitude, Michael C. Ferraro, and Peter D. Drummond. All authors have approved the final version of the manuscript and agree to be accountable for all aspects of the work. All authors report no conflicts of interest.

## Funding

The authors have nothing to report.

## Supporting information


**Data S1:** ejp70205‐sup‐0001‐app1.docx.

**Data S2:** ejp70205‐sup‐0002‐app2.docx.

**Data S3:** ejp70205‐sup‐0003‐app3.docx.

**Data S4:** ejp70205‐sup‐0004‐supinfo.docx.
