# Supplementary material for: Clinical Presentation of Shoulder‐Hand Syndrome: A Systematic Review
Source: Eur J Pain. 2026 Feb 4;30(2):e70205. doi: 10.1002/ejp.70205 (PMC12887613; doi:10.1002/ejp.70205)
Supplement: Supplementary file 1 — Data S1: ejp70205‐sup‐0001‐app1.docx. [file EJP-30-0-s004.docx]

**APPENDIX 1**

**Table A1: Included articles that reference Steinbrocker’s SHS criteria**

| **Title** | **1^st^ Author, Year** | **SHS Criteria** |
| --- | --- | --- |
| Cortisone in treatment of shoulder-hand syndrome following acute myocardial infarction | Russek, H.1953 | Steinbrocker’s 1947 (Steinbrocker, 1947) |
| Shoulder-hand syndrome following myocardial infarction with special reference to prognosis | Edeiken, J.1957 | Cites Steinbrocker’s 1947 publication |
| Shoulder-hand syndrome - It's recognition and management | Baer, R. 1966 | Cites Steinbrocker’s 1947 publication |
| Shoulder-hand syndrome in patients with intracranial neoplasms | Walker, J. 1983 | Cites Steinbrocker’s 1947 publication |
| Shoulder-hand syndrome following myocardial infarction; treatment by procaine block of the stellate ganglion | Swan, D. 1951 | Cites Steinbrocker’s 1948 publication (Steinbrocker et al., 1948) |
| Prevalence of the Shoulder-Hand Pain Syndrome in an Inpatient Stroke Rehabilitation Population: A Quantitative Cross-Sectional Study | Chalsen, G. 1987 | Cites Steinbrocker’s 1958 publication (Steinbrocker & Argyros, 1958) |
| Probable cervical midline epidural septum complicating the treatment of a patient with upper extremity sympathetically maintained pain | Valley, M. 1994 | Cites Steinbrocker’s 1958 publication |
| Metastatic gastric cancer presenting with shoulder-hand syndrome: a case report | Massarotti, M. 2008 | Cites Steinbrocker’s 1958 publication |
| Bilateral shoulder-hand syndrome associated with phenobarbital administration - A case report | Reddy, M. 1985 | Cites Steinbrocker’s 1968 publication (Steinbrocker, 1968) |
| Shoulder-hand syndrome in a hemiplegic population: a 5-year retrospective study | Davis, S. 1977 | References section of book written by Steinbrocker, published 1972 (Steinbrocker, 1972) |
| The Shoulder-Hand Syndrome: Historical Review with Observations on Seventy-Three Patients | Rosen, P. 1957 | Cites Steinbrocker’s 1947 and 1948 publications (Steinbrocker, 1947; Steinbrocker et al., 1948) |
| Sudeck's Atrophy and the Shoulder-hand Syndrome | Taylor, J. 1958 | Cites Steinbrocker’s 1947 and 1948 publications (Steinbrocker, 1947; Steinbrocker et al., 1948) |
| Some uncommon rheumatic syndromes/shoulder-hand syndrome | Thompson, R. 1961 | Cites Steinbrocker’s 1947 and 1948 publications (Steinbrocker, 1947; Steinbrocker et al., 1948) |
| The shoulder-hand syndrome after stroke: a prospective clinical trial | Braus, D. 1994 | References Steinbrocker's 1947 criteria (Steinbrocker, 1947), and Kozin's definition of "definite" RSD (Kozin et al., 1981) |
| The painful hemiplegic shoulder: effects of intra-articular triamcinolone acetonide | Dekker, J. 1997 | References Steinbrocker 1958 (Steinbrocker & Argyros, 1958), and Tepperman (Tepperman et al., 1984) to devise a scoring system wherein a relevant symptom is assigned 1 point (e.g. pain in shoulder and hand). |

**Table A1:** *Title, 1^st^ author, year published, and criteria used to determine SHS in 15/33 included studies*. These articles reference Steinbrocker’s SHS criteria (independently or alongside other authors) for their participant inclusion criteria.

**Table A2: Included articles that reference authors other than Steinbrocker for SHS criteria**

| **Title** | **1^st^ Author, Year** | **SHS Criteria** |
| --- | --- | --- |
| Treatment of shoulder-hand syndrome with griseofulvin | Cohen, A. 1960 | 'Adam's Physical Diagnosis; an introduction to clinical medicine’ (Dennette et al., 1974) |
| Role of biphosphonates and lymphatic drainage type Leduc in the complex regional pain syndrome (shoulder-hand syndrome) | Santamato, A. 2009 | Zyluk et al. (Zyluk, 2003) for SHS criteria (CRPS-SHS as described by the authors). |
| Functional orthosis in shoulder joint subluxation after ischaemic brain stroke to avoid post-hemiplegic shoulder-hand syndrome: a randomized clinical trial | Hartwig, M. 2012 | Point-based SHS scoring system based on Braus et al. (Braus et al., 1994). An additional measure of muscle strength was included in accordance with the Medical Research Council, rated no muscle activity (0) to normal power (5). |
| Timeliness of the analgesic effect of superficial needling on shoulder-hand syndrome after stroke | Wang, J. 2015 | References 'Rehabilitation and Assessment and Treatment of Stroke' (Liao & Zhu, 1996) |
| Clinical observation on the effect of acupuncture and moxibustion combined with rehabilitation training on post-stroke shoulder hand syndrome: 针灸并用结合康复训练治疗中风后肩手综合征临床疗效观察 | Pan, J. 2020 | References 'Rehabilitation and Assessment and Treatment of Stroke' (Liao & Zhu, 1996) |

**Table A2:** *Title, 1^st^ author, year published, and criteria used to determine SHS in 5/33 included studies.* These articles reference criteria other than Steinbrocker’s for their participant inclusion criteria.

**Table A3: Included articles that did not provide any reference for their SHS criteria**

| **Title** | **1^st^ Author, Year** | **Criteria** |
| --- | --- | --- |
| Recurrent Post-Infarctional Shoulder-Hand Syndrome — Report of a Case with Unusual Clinical Evolution | Kammerling, E. 1950 | Shoulder pain, along with pain, swelling, tenderness, discoloration, and skin atrophy in the affected hand. Osteoporosis in the hand. |
| Phenobarbital and the shoulder-hand syndrome | Van der Korst, J. 1966 | Pain and/or stiffness of the shoulder; diffuse swelling or atrophy of soft tissues of homolateral hand. |
| Holistic approach to shoulder-hand syndrome | Amick, L. 1966 | Pain, limited ROM, tenderness of the shoulder, and swelling, tenderness, tissue atrophy, flexion contractures and trophic changes in the hand. |
| Treatment of the shoulder-hand syndrome with corticosteroids | Mowat, A. 1974 | Pain, restricted movement, and stiffness in the shoulder, and swelling, stiffness, temperature increase, hyperhidrosis, discolouration and limited function in the hand. Flexion deformities in affected hand. |
| Shoulder-hand syndrome after laparoscopic sterilisation | Low, L. 1978 | Shoulder pain, restricted shoulder motion, swelling in the right arm and hand, increased hand temperature with hyperhidrosis, pain, and limited movement. Mild osteoporosis in shoulder. |
| Shoulder-hand syndrome and symmetrical arthralgia in patients with tubo-ovarian carcinoma | Taggart, A. 1984 | No shoulder pain, limited ROM and stiffness in the shoulder, and pain, swelling, discolouration and shiny skin on the hand. |
| Shoulder-hand syndrome in patients with ovarian carcinoma | McGill, P. 1985 | Pain, swelling and stiffness in the shoulder and hand. Flexion deformities in the hand. |
| Shoulder-hand syndrome in cervical spinal cord injury | Aisen, P. 1994 | Shoulder pain, and pain, swelling, discolouration and temperature changes of the affected limb. Additionally, trophic changes (shiny, atrophic skin and/or dystrophic nails) and radiographic abnormalities of the hand were described. |
| The sympathetic skin response in the shoulder-hand syndrome complicating tetraplegia | Aisen, M. 1995 | Diffuse upper extremity pain described as 'burning', swelling, osteoporosis and dystrophic skin and nail changes. |
| Shoulder-hand syndrome in hemiplegic patients: Temperature, sympathetic skin responses, and nerve latencies of the affected and nonaffected upper extremity | Hesse, S. 1995 | Painful shoulder at rest or during mobilization, swelling in the wrist and hand, vasomotor changes, and tenderness to palpation. |
| Shoulder-hand syndrome in neurosurgical patients treated with barbiturates. A long-term evaluation | Desantis, A. 2000 | Intense pain and function impairment affecting the shoulder or hand, or both; limited range of motion, tenderness, and sudomotor and neurovascular changes. |
| Protocol to prevent shoulder-hand syndrome after stroke | Kondo, I. 2001 | SHS symptoms were divided into division A, and division B - both division A items and 1 division B item would result in a diagnosis of SHS. Division A: Swelling of the finger (swelling index ≥1.07), shoulder pain at rest or with mobilization. Division B: Finger pain, tenderness to palpation, and erythema of the hand or finger. |
| A shoulder-hand syndrome revealing a lung cancer | Akasabi, N. 2010 | Pain and limited function in the shoulder, and pain, hyperhidrosis, oedema and loss of function in the hand. |

**Table A3:** *Title, 1^st^ author, year published, and criteria used to determine SHS in 13/33 included studies.* These articles are categorised here as they are not referencing any specific source of SHS criteria used for inclusion of participants.
