# Supplementary material for: Clinical Presentation of Shoulder‐Hand Syndrome: A Systematic Review
Source: Eur J Pain. 2026 Feb 4;30(2):e70205. doi: 10.1002/ejp.70205 (PMC12887613; doi:10.1002/ejp.70205)
Supplement: Supplementary file 2 — Data S2: ejp70205‐sup‐0002‐app2.docx. [file EJP-30-0-s003.docx]

**Appendix 2**

This appendix displays the results of an additional search of studies describing clinical characteristics of complex *regional pain syndrome (CRPS) after stroke* in order to compare the reported patient phenotypes with those described in the already identified studies on *shoulder hand syndrome (SHS)*.

An electronic database search was performed on PubMed for articles published between 2000 and 2025. The final search was conducted in October 2025. Keywords and phrases used in this search included ‘post-stroke complex regional pain syndrome,’ post-stroke CRPS,’ complex regional pain syndrome after stroke,’ and ‘CRPS after stroke.’ As with the original search, articles were included if they described direct contact with participants or participant data (e.g. case studies, retrospective studies); reviews, systematic reviews, and meta-analyses were excluded. To be included, the article needed to clearly detail the symptoms of CRPS of the upper limb after stroke. Articles that used the above CRPS-related terms and additionally referred to ‘shoulder hand syndrome’ or to retired terms of CRPS such as ‘reflex sympathetic dystrophy’ were also included. All articles identified through PubMed were retrieved and full texts screened by one reviewer (RM). A second reviewer (AG) screened a percentage (20%) of the selected articles to confirm eligibility. The screening process is displayed in Fig. A1 with a flow diagram. A total of 305 articles were identified in the search. After a full-text review of these manuscripts, twenty-eight were included in this additional review (flow chart). The study characteristics are shown in table A4. The reported shoulder symptoms for each article are displayed below in table A5.

Of these twenty-eight articles, twenty-two utilise CRPS criteria (Akhavan Hejazi & Mazlan, 2012; Albayrak et al., 2016; Altas et al., 2020; Anandkumar & Manivasagam, 2014; Do et al., 2022; Eun Young et al., 2016; Glize et al., 2022; Han et al., 2014; Katsura et al., 2022; Kim et al., 2024; Kim et al., 2025; Kim et al., 2020; Kim et al., 2016; Lai et al., 2008; Lee et al., 2018; Park et al., 2007; Qiu et al., 2025; Riedl et al., 2001; Ryu et al., 2024; Topcuoglu et al., 2015; Xie et al., 2025; Yoo et al., 2012), two apply RSD criteria (Ersoz et al., 2006; Petchkrua et al., 2000), two used SHS criteria (Daviet et al., 2004; Kalita et al., 2016), the remaining two articles used a combination of CRPS and RSD (Matayoshi et al., 2009), or CRPS and SHS criteria (Yavuz Keleş et al., 2020). Many articles also included three-phase bone scintigraphy as part of their criteria, which is not part of the widely accepted IASP Budapest criteria for CRPS (Harden et al., 2007). Only nine of these included articles reported shoulder symptoms highlighting that most authors using these search terms in their studies either included a different set of patients from those included in the SHS studies, or that they paid no particular attention to shoulder complaints. Of the nine studies 44.4% (n = 4) utilised CRPS criteria, 22.2% (n = 2) applied RSD criteria, and 22.2% (n = 2) employed SHS criteria; the remaining two studies use a combination of either CRPS and SHS (n = 1) or CRPS and RSD criteria (n = 1). Interestingly, most of these papers, except the earlier ones, are not published in countries that usually produce CRPS research (D’Souza et al., 2025). It may be that studies from countries that more frequently publish CRPS research, e.g. the UK or USA (Berk et al., 2025; Coggins et al.; Illescas et al., 2025; Smith & Miranda, 2025), do not use the term *CRPS after stroke* or *post-stroke CRPS.*

Compared to SHS patients described in the reviewed papers in our main search, the described shoulder symptoms in ‘CRPS after stroke’ are predominantly shoulder pain and limited ROM. There are no reports of *shoulder stiffness*, or *osteopenia/osteoporosis on X-ray* in these CRPS cohorts. As most of the included articles rely on a version of the IASP criteria, which do not specify shoulder symptoms as a criterion for CRPS, these symptoms may have been present but not reported. Alternatively, absence of such reported signs and symptoms may further support the possibility that SHS and CRPS after-stroke are distinct conditions; further research in patients developing CRPS-like limb conditions following stroke will be required to clarify these issues.


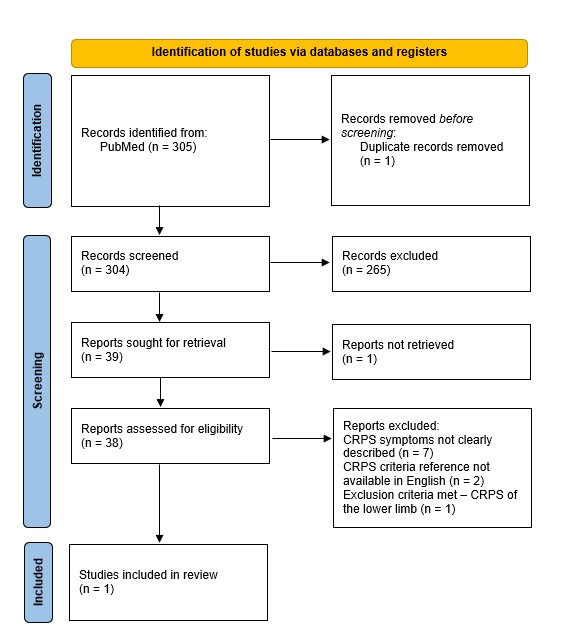


Fig. A1 PRISMA Flowchart of study screening and selection

| **Table A4: Study characteristics** | | | | |  | |  | |  |
| --- | --- | --- | --- | --- | --- | --- | --- | --- | --- |
|  | | | | | **Age** | | **Sex** | |  |
| **First author** | **Year** | **Region** | **Study design** | **Sample size** | **Mean** | **Range** | **Male** | **Female** | **Diagnostic criteria for CRPS** |
| Petchkrua, W** | 2000 | USA | Cross-sectional | 64 | NR | 40-81 | 31 | 33 | RSD criteria based on a pre-1994 scoring sheet published by Gibbons (Gibbons & Wilson, 1992), additionally only TPBS positive in affected limb |
| Riedl, B | 2001 | Germany | Case-control | 38 | NR | 26-84 | 12 | 26 | CRPS criteria from Harden 1999 (Harden et al., 1999) |
| Daviet, J.C** | 2004 | France | Case series | 42 | 62.5 | 37-84 | 18 | 24 | Post-stroke SHS criteria from Steinbrocker 1958 (Geurts et al., 2000; Steinbrocker & Argyros, 1958) |
| Ersoz, M** | 2006 | India | Case-control | 69 | 60 | 33-77 | 43 | 26 | RSD criteria based on Kozin 1985 (Kozin et al., 1981) |
| Park, S | 2007 | Korea | Cohort | 38 | 52.15 | NR | 25 | 13 | CRPS criteria from Stanton-Hicks 1995 (Stanton-Hicks et al., 1995), additionally only TPBS positive in lower arm/hand |
| Lai, M.H | 2008 | Taiwan | Case report | 1 | 64 | NR | 1 | 0 | Cites Turk 1987 as basis for CRPS criteria (Turk & Rudy, 1987) |
| Matayoshi, S** | 2009 | Japan | Case-control | 59 | 69.1 | NR | 38 | 21 | IASP 1994 CRPS type I criteria, all patients met IASP criteria as defined by Veldman 1993 (Pain, 1994; Veldman et al., 1993) |
| Han, E | 2012 | Korea | Retrospective case-control | 252 | 62.3 | NR | 141 | 110 | CRPS criteria from Harden 1999 (Harden et al., 1999), additionally must be TPBS positive in lower arm |
| Hejazi, S** | 2012 | Malaysia | Case report | 1 | 66 | NR | 1 | 0 | No CRPS criteria cited, clinical signs of CRPS type I similar to Budapest criteria |
| Yoo, S | 2012 | Korea | Prospective cohort | 42 | 60.2 | NR | 25 | 19 | Budapest CRPS criteria (Harden et al., 2010) |
| Anandkumar, S | 2013 | India | Case report | 1 | 48 | NR | 0 | 1 | Budapest CRPS criteria (Harden et al.) |
| Topcuoglu, A | 2015 | Turkey | Prospective cohort | 40 | 66.7 | 35-80 | 22 | 18 | Budapest CRPS type I Criteria (Harden et al., 2010; Harden et al., 2007) |
| Albayrak, I** | 2016 | Turkey | Case series | 2 | 58.5 | 48-69 | 0 | 2 | Budapest CRPS criteria (Harden et al., 2007) |
| Kalita, J** | 2016 | India | RCT | 58 | 55.15 | 35-85 | 34 | 24 | SHS criteria from Braus 1994 (Braus et al., 1994), based on Steinbrocker (Steinbrocker & Argyros, 1958) |
| Kim, Y | 2016 | Korea | Prospective cohort | 23 | 64.4 | NR | 11 | 12 | Budapest CRPS criteria (Harden et al., 2005), additionally must be TPBS positive in affected arm/hand, further confirmation via ultrasonography of wrist tendons |
| Young, H | 2016 | Korea | RCT | 21 | 65.2 | 44-77 | 11 | 10 | Budapest CRPS criteria (Harden et al., 2007), additionally must be TPBS positive |
| Lee, A** | 2018 | Korea | Case report | 1 | 72 | NR | 0 | 1 | Budapest CRPS criteria (Harden et al., 2005), additionally must be TPBS positive in upper limb |
| Keleş, B** | 2020 | Turkey | Case-control | 40 | 63.2 | 40-75 | 27 | 13 | Budapest CRPS type I criteria (Harden et al., 2010), CRPS severity score based on SHS criteria by Braus (Braus et al., 1994; Steinbrocker & Argyros, 1958) |
| Kim, J** | 2020 | Korea | Retrospective case-control | 80 | 63 | NR | 45 | 35 | Budapest CRPS criteria (Harden et al., 2007), additionally required positive TPBS in the limbs |
| Umay, E | 2020 | Turkey | Retrospective case-control | 426 | 67 | NR | 216 | 256 | Budapest CRPS type I criteria (Harden et al., 2010) |
| Do, J | 2022 | Korea | Retrospective cross-sectional | 313 | 65.5 | NR | 161 | 152 | No CRPS criteria cited, clinical signs of CRPS type I similar to Budapest criteria. Symptoms confirmed with positive TPBS |
| Glize, B** | 2022 | France | RCT | 76 | 68.3 | NR | 49 | 27 | Budapest CRPS criteria (Harden et al., 2010) |
| Katsura, Y | 2022 | Japan | Cross-sectional | 102 | 72.1 | NR | 44 | 58 | Budapest CRPS criteria (Harden et al., 2010) |
| Kim, C | 2024 | Korea | Retrospective case-control | 141 | 68.2 | NR | 83 | 58 | Budapest CRPS type 1 criteria (Harden et al., 2007), additionally must be TPBS positive in upper limb |
| Ryu, J. W | 2024 | Korea | Retrospective cohort | 7 | 65.9 | NR | 4 | 3 | Budapest CRPS criteria (Harden et al., 2010), additionally must be TPBS positive |
| Qui, X | 2025 | China | RCT | 53 | 66.1 | 18-85 | 36 | 17 | Budapest CRPS type 1 criteria (Harden et al., 2007), additionally severity of CRPS was recorded, based on Harden 2017 (Harden et al., 2017) |
| Kim, C | 2025 | Korea | Retrospective case-control | 226 | 68.3 | NR | 129 | 97 | Budapest CRPS type 1 criteria (Harden et al., 2007), additionally must be TPBS positive in upper limb |
| Xie, Q | 2025 | China | Retrospective case-control | 376 | 62.5 | NR | 182 | 82 | Budapest CRPS criteria (Harden et al., 2007) |
| **Table A4:** Study characteristics for all included articles. **Indicates that the authors reported shoulder symptoms | | | | | | | | | |

| **Table A5: CRPS after stroke shoulder symptoms reported across included articles** | | | | | | | |
| --- | --- | --- | --- | --- | --- | --- | --- |
|  | | | | | **Pain and limited ROM associated with specific movement** | | |
| **First author** | **Sample size** | **Pain (%)** | **Limited ROM (%)** | **Tenderness** | **Humeral abduction (%)** | **Flexion rotation (%)** | **External rotation (%)** |
| Petchkrua, W | 64 | R | R | -- | -- | -- | -- |
| Riedl, B | 38 | -- | -- | -- | -- | -- | -- |
| Daviet, J.C | 42 | R | R | R | R | -- | R |
| Ersoz, M | 69 | 100 | 100 | -- | -- | -- | -- |
| Park, S | 38 | -- | -- | -- | -- | -- | -- |
| Lai, M.H | 1 | -- | -- | -- | -- | -- | -- |
| Matayoshi, S | 59 | 39 | 39 | -- | 39 | 39 | -- |
| Han, E | 252 | -- | -- | -- | -- | -- | -- |
| Hejazi, S* | 1 | 100 | 100 | -- | -- | -- | -- |
| Yoo, S | 42 | -- | -- | -- | -- | -- | -- |
| Anandkumar, S | 1 | -- | -- | -- | -- | -- | -- |
| Topcuoglu, A | 40 | -- | -- | -- | -- | -- | -- |
| Albayrak, I* | 2 | -- | 100 | -- | -- | -- | -- |
| Kalita, J | 58 | R | R | -- | R | R | R |
| Kim, Y | 23 | -- | -- | -- | -- | -- | -- |
| Young, H | 21 | -- | -- | -- | -- | -- | -- |
| Lee, A* | 1 | -- | -- | -- | 100 | 100 | 100 |
| Keleş, B | 40 | R | R | -- | -- | -- | -- |
| Kim, J | 80 | 16 | -- | -- | -- | -- | -- |
| Umay, E | 426 | -- | -- | -- | -- | -- | -- |
| Do, J | 313 | -- | -- | -- | -- | -- | -- |
| Glize, B | 76 | R | -- | -- | -- | -- | -- |
| Katsura, Y | 102 | -- | -- | -- | -- | -- | -- |
| Kim, C | 141 | -- | -- | -- | -- | -- | -- |
| Ryu, J. W | 7 | -- | -- | -- | -- | -- | -- |
| Qui, X | 53 | -- | -- | -- | -- | -- | -- |
| Kim, C | 226 | -- | -- | -- | -- | -- | -- |
| Xie, Q | 376 | -- | -- | -- | -- | -- | -- |
| **Table A5.** Shoulder symptoms reported across all included articles. Numbers represent the percentage of participants for whom the symptom was recorded. – indicates that the symptom was not reported. *R* indicates that the symptom was reported, but no data were available on the percentage of patients that displayed that symptom. *n = ≤2 | | | | | | | |

**Reference List**

Akhavan Hejazi, S. M., & Mazlan, M. (2012). Concurrent peripheral pathologies and complex regional pain syndrome type 1 as contributors to acute post-stroke shoulder pain: a case report. Acta Med Iran, 50(4), 292-294.

Albayrak, I., Apiliogullari, S., Onal, O., Gungor, C., Saltali, A., & Levendoglu, F. (2016). Pulsed radiofrequency applied to the dorsal root ganglia for treatment of post-stroke complex regional pain syndrome: A case series. J Clin Anesth, 33, 192-197. https://doi.org/10.1016/j.jclinane.2016.03.059

Altas, E. U., Onat, Ş., Konak, H. E., & Polat, C. S. (2020). Post-stroke complex regional pain syndrome and related factors: Experiences from a tertiary rehabilitation center. J Stroke Cerebrovasc Dis, 29(9), 104995. https://doi.org/10.1016/j.jstrokecerebrovasdis.2020.104995

Anandkumar, S., & Manivasagam, M. (2014). Multimodal physical therapy management of a 48-year-old female with post-stroke complex regional pain syndrome. Physiother Theory Pract, 30(1), 38-48. https://doi.org/10.3109/09593985.2013.814186

Berk, A., Good, L., Burkhart, R., Jawanda, H., Florentino, S., & Napora, J. (2025). Patients with vitamin D deficiency are more likely to develop complex region pain syndrome after extremity fractures: a large database propensity-matched cohort study. European Journal of Orthopaedic Surgery & Traumatology, 35(1), 382. https://doi.org/10.1007/s00590-025-04515-x

Braus, D. F., Krauss, J. K., & Strobel, J. (1994). The shoulder-hand syndrome after stroke: a prospective clinical trial. Ann Neurol, 36(5), 728-733. https://doi.org/10.1002/ana.410360507

Coggins, J., McCabe, C., Walsh, N., Pearson, J., Rolls, C., & Llewellyn, A. Identifying recommendations to improve therapy-led management of complex regional pain syndrome in England. British Journal of Pain, 0(0), 20494637251389063. https://doi.org/10.1177/20494637251389063

D’Souza, R. S., Klasova, J., Saini, C., Chang, A., Music, S., Shah, J. D., Elmati, P. R., Chitneni, A., To, J., Prokop, L. J., & Hussain, N. (2025). Global Burden of Complex Regional Pain Syndrome in At-Risk Populations: Estimates of Prevalence From 35 Countries Between 1993 and 2023. Anesthesia & Analgesia. https://journals.lww.com/anesthesia-analgesia/fulltext/9900/global_burden_of_complex_regional_pain_syndrome_in.1163.aspx

Daviet, J. C., Dudognon, P., Preux, P. M., Rebeyrotte, I., Lacroix, P., Munoz, M., & Salle, J. Y. (2004). Reliability of transcutaneous oxygen tension measurement on the back of the hand and complex regional pain syndrome after stroke. Arch Phys Med Rehabil, 85(7), 1102-1105. https://doi.org/10.1016/j.apmr.2003.09.034

Do, J. G., Choi, J. H., Park, C. H., Yoon, K. J., & Lee, Y. T. (2022). Prevalence and Related Factors for Poststroke Complex Regional Pain Syndrome: A Retrospective Cross-Sectional Cohort Study. Arch Phys Med Rehabil, 103(2), 274-281. https://doi.org/10.1016/j.apmr.2021.08.003

Ersoz, M., Inanir, M., & Kurtaran, A. (2006). Sympathetic skin responses in hemiplegic patients with and without complex regional pain syndrome [Article]. Neurology India, 54. https://link-gale-com.liverpool.idm.oclc.org/apps/doc/A150779200/AONE?u=livuni&amp;sid=bookmark-AONE&amp;xid=ce0767b3

Eun Young, H., Hyeyun, K., & Sang Hee, I. (2016). Pamidronate effect compared with a steroid on complex regional pain syndrome type I: Pilot randomised trial. Neth J Med, 74(1), 30-35.

Geurts, A., Visschers, B. A. J. T., Limbeek, J., & Ribbers, G. (2000). Systematic review of aetiology and treatment of post-stroke hand oedema and shoulder-hand syndrome. Scandinavian journal of rehabilitation medicine, 32, 4-10.

Gibbons, J. J., & Wilson, P. R. (1992). RSD score: criteria for the diagnosis of reflex sympathetic dystrophy and causalgia. Clin J Pain, 8(3), 260-263.

Glize, B., Cook, A., Benard, A., Sagnier, S., Olindo, S., Poli, M., Debruxelles, S., Renou, P., Rouanet, F., Bader, C., Dehail, P., & Sibon, I. (2022). Early multidisciplinary prevention program of post-stroke shoulder pain: A randomized clinical trial. Clin Rehabil, 36(8), 1042-1051. https://doi.org/10.1177/02692155221098733

Han, E. Y., Jung, H. Y., & Kim, M. O. (2014). Absent median somatosensory evoked potential is a predictor of type I complex regional pain syndrome after stroke. Disabil Rehabil, 36(13), 1080-1084. https://doi.org/10.3109/09638288.2013.829530

Harden, N. R., Bruehl, S., Perez, R., Birklein, F., Marinus, J., Maihofner, C., Lubenow, T., Buvanendran, A., Mackey, S., Graciosa, J., Mogilevski, M., Ramsden, C., Chont, M., & Vatine, J. J. (2010). Validation of proposed diagnostic criteria (the "Budapest Criteria") for Complex Regional Pain Syndrome. Pain, 150(2), 268-274. https://doi.org/10.1016/j.pain.2010.04.030

Harden, R., Bruehl, S., Wilson, P., Stanton-Hicks, M., & Harden, R. (2005). CRPS: Current diagnosis and therapy. Progress in Pain Research and Management, 32, 45-58.

Harden, R. N., Bruehl, S., Galer, B. S., Saltz, S., Bertram, M., Backonja, M., Gayles, R., Rudin, N., Bhugra, M. K., & Stanton-Hicks, M. (1999). Complex regional pain syndrome: are the IASP diagnostic criteria valid and sufficiently comprehensive? Pain, 83(2), 211-219. https://doi.org/10.1016/s0304-3959(99)00104-9

Harden, R. N., Bruehl, S., Stanton-Hicks, M., & Wilson, P. R. (2007). Proposed New Diagnostic Criteria for Complex Regional Pain Syndrome. Pain Medicine, 8(4), 326-331. https://doi.org/10.1111/j.1526-4637.2006.00169.x

Harden, R. N., Maihofner, C., Abousaad, E., Vatine, J. J., Kirsling, A., Perez, R., Kuroda, M., Brunner, F., Stanton-Hicks, M., Marinus, J., van Hilten, J. J., Mackey, S., Birklein, F., Schlereth, T., Mailis-Gagnon, A., Graciosa, J., Connoly, S. B., Dayanim, D., Massey, M., . . . Bruehl, S. (2017). A prospective, multisite, international validation of the Complex Regional Pain Syndrome Severity Score. Pain, 158(8), 1430-1436. https://doi.org/10.1097/j.pain.0000000000000927

Illescas, A., Chen, T., Chan, W., Richman, D., Memtsoudis, S., Sideris, A., Poeran, J., & Gungor, S. (2025). OP31 Yearly incidence of complex regional pain syndrome in adult and pediatric patients in the United States: an analysis using the merative marketscan database. Regional Anesthesia &amp;amp; Pain Medicine, 50(Suppl 1), A21. https://doi.org/10.1136/rapm-2025-ESRA.31

Kalita, J., Misra, U., Kumar, A., & Bhoi, S. K. (2016). Long-term Prednisolone in Post-stroke Complex Regional Pain Syndrome. Pain Physician, 19(8), 565-574.

Katsura, Y., Ohga, S., Shimo, K., Hattori, T., Yamada, T., & Matsubara, T. (2022). Post-Stroke Complex Regional Pain Syndrome and Upper Limb Inactivity in Hemiplegic Patients: A Cross-Sectional Study. J Pain Res, 15, 3255-3262. https://doi.org/10.2147/jpr.S379840

Kim, C. Y., Choi, S. B., & Lee, E. S. (2024). Prevalence and predisposing factors of post-stroke complex regional pain syndrome: Retrospective case-control study. J Stroke Cerebrovasc Dis, 33(2), 107522. https://doi.org/10.1016/j.jstrokecerebrovasdis.2023.107522

Kim, C. Y., Kim, Y. B., & Lee, E. S. (2025). Risk factors analysis of post-stroke complex regional pain syndrome in patients with first-ever subacute stroke. Clin Neurol Neurosurg, 257, 109035. https://doi.org/10.1016/j.clineuro.2025.109035

Kim, J. Y., Yoon, S. Y., Kim, J., Jeong, Y. H., & Kim, Y. W. (2020). Neural substrates for poststroke complex regional pain syndrome type I: a retrospective case-control study using voxel-based lesion symptom mapping analysis. Pain, 161(6), 1311-1320. https://doi.org/10.1097/j.pain.0000000000001816

Kim, Y. W., Kim, Y., Kim, J. M., Hong, J. S., Lim, H. S., & Kim, H. S. (2016). Is poststroke complex regional pain syndrome the combination of shoulder pain and soft tissue injury of the wrist?: A prospective observational study: STROBE of ultrasonographic findings in complex regional pain syndrome. Medicine (Baltimore), 95(31), e4388. https://doi.org/10.1097/md.0000000000004388

Kozin, F., Ryan, L. M., Carerra, G. F., Soin, J. S., & Wortmann, R. L. (1981). The reflex sympathetic dystrophy syndrome (RSDS). III. Scintigraphic studies, further evidence for the therapeutic efficacy of systemic corticosteroids, and proposed diagnostic criteria. Am J Med, 70(1), 23-30. https://doi.org/10.1016/0002-9343(81)90407-1

Lai, M. H., Wang, T. Y., Chang, C. C., Li, T. Y., & Chang, S. T. (2008). Cerebellar diaschisis and contralateral thalamus hyperperfusion in a stroke patient with complex regional pain syndrome. J Clin Neurosci, 15(10), 1166-1168. https://doi.org/10.1016/j.jocn.2007.06.017

Lee, A., Jung, Y., Kwon, H. K., & Pyun, S. B. (2018). Complex Regional Pain Syndrome of Non-hemiplegic Upper Limb in a Stroke Patient: A Case Report. Ann Rehabil Med, 42(1), 175-179. https://doi.org/10.5535/arm.2018.42.1.175

Matayoshi, S., Shimodozono, M., Hirata, Y., Ueda, T., Horio, S., & Kawahira, K. (2009). Use of calcitonin to prevent complex regional pain syndrome type I in severe hemiplegic patients after stroke. Disabil Rehabil, 31(21), 1773-1779. https://doi.org/10.1080/09638280902795573

Pain, I. A. f. t. S. o. (1994). Classification of Chronic Pain (H. Merskey & N. Bogduk, Eds. 2nd ed.). IASP Press.

Park, S. G., Hyun, J. K., Lee, S. J., & Jeon, J. Y. (2007). Quantitative evaluation of very acute stage of complex regional pain syndrome after stroke using three-phase bone scintigraphy. Nucl Med Commun, 28(10), 766-770. https://doi.org/10.1097/MNM.0b013e32828e513f

Petchkrua, W., Weiss, D. J., & Patel, R. R. (2000). Reassessment of the incidence of complex regional pain syndrome type 1 following stroke. Neurorehabil Neural Repair, 14(1), 59-63. https://doi.org/10.1177/154596830001400107

Qiu, X., Gao, T., Hua, Y., Zhang, Y., Zhang, A., & Bai, Y. (2025). Efficacy of manual lymphatic drainage combined with repetitive transcranial magnetic stimulation in post-stroke complex regional pain syndrome: a pilot study. Disabil Rehabil, 47(12), 3115-3123. https://doi.org/10.1080/09638288.2024.2416052

Riedl, B., Beckmann, T., Neundörfer, B., Handwerker, H. O., & Birklein, F. (2001). Autonomic failure after stroke--is it indicative for pathophysiology of complex regional pain syndrome? Acta Neurol Scand, 103(1), 27-34. https://doi.org/10.1034/j.1600-0404.2001.00139.x

Ryu, J. W., Hwang, I. S., & Lim, S. K. (2024). Use of Bioelectrical Impedance Analysis to Explore the Effectiveness of Stellate Ganglion Block in Patients with Post-Stroke Complex Regional Pain Syndrome: A Retrospective Pilot Study. J Pers Med, 14(3). https://doi.org/10.3390/jpm14030258

Smith, A. C., & Miranda, B. H. (2025). Lived experience perspectives of persons with complex regional pain syndrome: a survey study of the history of their condition, treatments and functional outcomes. British Journal of Pain, 19(4), 274-284. https://doi.org/10.1177/20494637251336636

Stanton-Hicks, M., Jänig, W., Hassenbusch, S., Haddox, J. D., Boas, R., & Wilson, P. (1995). Reflex sympathetic dystrophy: changing concepts and taxonomy. Pain, 63(1), 127-133. https://doi.org/10.1016/0304-3959(95)00110-e

Steinbrocker, O., & Argyros, T. G. (1958). The Shoulder-Hand Syndrome: Present Status as a Diagnostic and Therapeutic Entity. Medical Clinics of North America, 42(6), 1533-1553. https://doi.org/https://doi.org/10.1016/S0025-7125(16)34203-1

Topcuoglu, A., Gokkaya, N. K., Ucan, H., & Karakuş, D. (2015). The effect of upper-extremity aerobic exercise on complex regional pain syndrome type I: a randomized controlled study on subacute stroke. Top Stroke Rehabil, 22(4), 253-261. https://doi.org/10.1179/1074935714z.0000000025

Turk, D. C., & Rudy, T. E. (1987). IASP taxonomy of chronic pain syndromes: preliminary assessment of reliability. Pain, 30(2), 177-189. https://doi.org/10.1016/0304-3959(87)91073-6

Veldman, P. H., Reynen, H. M., Arntz, I. E., & Goris, R. J. (1993). Signs and symptoms of reflex sympathetic dystrophy: prospective study of 829 patients. Lancet, 342(8878), 1012-1016. https://doi.org/10.1016/0140-6736(93)92877-v

Xie, Q., Song, Q., Deng, J., Cheng, X., Xue, A., & Luo, S. (2025). Development and validation of a nomogram to predict the risk of post-stroke complex regional pain syndrome. Front Aging Neurosci, 17, 1577256. https://doi.org/10.3389/fnagi.2025.1577256

Yavuz Keleş, B., Önder, B., Kesiktaş, F. N., Öneş, K., & Paker, N. (2020). Acute effects of contrast bath on sympathetic skin response in patients with poststroke complex regional pain syndrome. Somatosens Mot Res, 37(4), 320-325. https://doi.org/10.1080/08990220.2020.1830756

Yoo, S. D., Jung, S. S., Kim, H. S., Yun, D. H., Kim, D. H., Chon, J., & Hong, D. W. (2012). Efficacy of ultrasonography guided stellate ganglion blockade in the stroke patients with complex regional pain syndrome. Ann Rehabil Med, 36(5), 633-639. https://doi.org/10.5535/arm.2012.36.5.633
