# Supplementary material for: Clinical Presentation of Shoulder‐Hand Syndrome: A Systematic Review
Source: Eur J Pain. 2026 Feb 4;30(2):e70205. doi: 10.1002/ejp.70205 (PMC12887613; doi:10.1002/ejp.70205)
Supplement: Supplementary file 3 — Data S3: ejp70205‐sup‐0003‐app3.docx. [file EJP-30-0-s001.docx]

**Appendix 3**

**Table A6: Time course of SHS symptoms**

| **1^st^ Author** | **Year** | **Sample size** | **Associated condition** | **Time course of SHS development** | **Follow-up** |
| --- | --- | --- | --- | --- | --- |
| Kammerling, E | 1950 | 1 | Myocardial infarction | SHS symptoms onset 5 weeks after myocardial infarct. Presented with severe shoulder and hand pain, developed over the next four months to include pain and swelling in both hands. Progressed to Stage II as described by Steinbrocker – swelling in hands subsided but pronounced stiffness and flexion deformity was observed for 2-3 months. Patient then advanced to third stage two months later (after a second myocardial infarction), and pain and swelling in the hands returned, and did not improve. These symptoms continued for six months until observation ended. | No further follow-up after the final six months |
| Swan, D | 1951 | 3 | Myocardial infarction | **Case 1:** Symptoms arose 5 weeks after a heart attack, continued, and got worse for 6 months – patient then received a stellate ganglion block (SBG). Symptoms significantly improved 1 month later; this improvement was sustained for 2 years until follow-up ended. [Still some mild residual symptoms]  **Case 2**: Symptoms arose 2 months after a heart attack; continued for 2 months before patient received SGB. After three weeks, symptoms were mostly improved (apart from some aching and stiffness in the fingers) until his death 11 months later.  **Case 3:** Symptoms started 3 weeks after myocardial infarction and progressed until 2 months later (entered stage II); pain and swelling reduced but disability worse. Entered stage III 6 months later (confirmed by X-ray), patient reports significant pain in shoulder and hand. After receiving SGB, symptoms were improved and somewhat resolved 6 months later when follow-up ended. | 6 months – 2 years  All patients reported at least some residual symptoms, predominantly reduced movement and function in the affected hand |
| Russek, H | 1953 | 17 | Myocardial infarction | [*Only three cases reported in detail*] The mean elapsed interval between the onset of myocardial infarction and development of SHS syndrome was 6.9 weeks (range 4-18 weeks). Time between recognition of SHS and application of cortisone therapy was 6.5 weeks.  **Case 1**: 8 weeks after myocardial infarction, patient exhibited pain in shoulder and arms. 5 months after the heart attack, symptoms developed to limited ROM in the shoulder and hands, and early flexion contractures in the digits. The affected hand was cold and showed signs of atrophy of subcutaneous tissue. A diagnosis of SHS was given. Cortisone therapy was prescribed for two months. Patient experienced “relief of all discomfort” and ROM in the shoulder was improved. 18 months after cortisone was stopped, further improvement was noted, though mild flexion contracture in the fingers persisted.  **Case 2:** Pain and limited ROM in the shoulder developed 4 months after myocardial infarction. Symptoms worsened over next 2 months to include severe pain and immobility of the shoulder; the hand exhibited increased blood flow and was swollen and painful. 24 hours after cortisone therapy, pain was relieved, within 48 hours almost all symptoms were resolved. Length of follow-up unknown, but symptom resolution was sustained, with only moderate restriction in abduction at the shoulder remaining.  **Case 3**: 1 month after myocardial infarction, patient reported discomfort in the left shoulder. 1 month later, this progressed to severe pain and complete restriction of abduction in the shoulder. That same month, cortisone treatment started. 2 weeks later, the shoulder pain was “tolerable” but ROM in the shoulder was still significantly restricted. After an additional week of cortisone [3 weeks total], pain was resolved and normal range of motion returned to the shoulder. 9 months later, at the end of follow-up, the patient had no recurrence of symptoms. | 18 weeks – 9 months  Symptoms were improved after cortisone therapy, but some mild hand and shoulder symptoms persisted for 2/3 cases |
| Edeiken, J | 1957 | 42 | Myocardial infarction | The earliest onset of SHS occurred 2 weeks after a myocardial infarction, the majority of patients developed symptoms within 4 months. Two cases occurred 5 months after the inciting event, four 6 months after, and the longest interval between infarct and SHS was 14 months. Most patients (29) obtained complete relief within 8 months of the onset of SHS. Nine other patients (including one that suffered two infarcts) had no symptoms after 10-17 months. One case persisted for 22 months, and the longest duration of symptoms was 2 years (2 patients). Only 5/42 patients reached the third stage of SHS as described by Steinbrocker. | No further follow-up reported |
| Rosen, P | 1957 | 73 | Several | The time interval between the onset of associated condition (which varied between patients) and development of SHS ranged from 24 hours – 4 years. | No follow-up reported |
| Taylor, J | 1958 | 6 | Several | Four cases were associated with injury to the shoulder, three were resolved within 6 months, the remaining was reported to still have persistent symptoms and significant disability after 2 years. One case (following myocardial infarction) was resolved within an unknown time frame. The final case (idiopathic) had a “slight residual pain or impairment of function" after 2 years. | No further follow-up reported |
| Cohen, A | 1960 | 12 | Several | [*Only three cases reported in detail*] **Case 1:** (Idiopathic) First symptoms, shoulder and hand pain, appeared in February. Nine months later, symptoms progressed, worsening pain in the shoulder, pain and swelling in the hand, and very limited ROM in shoulder and digits. After 2 weeks of Griseofulvin, shoulder and hand symptoms improved, but residual limited ROM and swelling in hands persisted. Three weeks later, shoulder symptoms were improved, but hand symptoms persisted  **Case 2:** (Myocardial infarction) Patient reported having pain in the shoulder and hand, with swelling and limited ROM in the fingers for 6 months. After 1 week of Griseofulvin, pain in shoulder and hand was resolved, and hand swelling was reduced by 50%. After a further “few weeks” of treatment, the patient was free of hand and shoulder symptoms.  **Case 3:** (Idiopathic) Patient exhibited pain in shoulder and hand, accompanied by hand swelling and limited ROM in arm/shoulder movement. These symptoms developed gradually over the course of 1 year. After Griseofulvin treatment (for an unspecified amount of time), the patients’ symptoms were “much improved.” | No follow-up reported |
| Thompson, R | 1961 | 17 | Several | Not reported | No follow-up reported |
| Amick, L | 1966 | 21 | Psychological | [*Only three cases reported in detail*] **Case 3:** Shoulder pain developed a week after the patient injured their shoulder. Symptoms persisted and worsened for 8 weeks, until a course of psychological treatment was started. A few weeks later symptoms were resolved.  **Case 9:** Shoulder and hand symptoms had developed and progressed over 7 years, after the death of their spouse and loss of their job. Patient undertook psychological counselling – no information available if symptoms persisted or were resolved.  **Case 19:** No single inciting incident, several years of general unhappiness and psychological trauma. The patient presented with severe hand and shoulder symptoms (such as significant flexion contractures in affected hand). Patient undertook physical and psychological therapy, and is implied to have improved, though no specific information was provided. | No follow-up reported |
| Baer, R | 1966 | 3 | Myocardial, injury, idiopathic | **Case 1:** (Myocardial infarction): Myocardial infarction 6 months before onset of shoulder pain, hand symptoms develop over following 2 months. After 10 days of treatment (including physical therapy) ROM in the shoulder was significantly improved. Follow-up “at intervals” showed reduction (but not complete resolution) of pain in the shoulder, reversal of contractures in the hand, and further increased ROM in the shoulder.  **Case 2:** (Arthritis/trauma): Wrist fracture 4 months before hospital admission, symptoms developed over this time. Patient presented with severe shoulder and hand symptoms. After 1 month of physical therapy, ROM in the shoulder was significantly improved, and only minimal pain persisted. Follow-up 2 months later revealed symptoms were mostly resolved.  **Case 3:** (Idiopathic) Patient reported acute onset of chest pain and joint pain 3 months before hospitalisation. Amount of time in hospital not detailed, but the patient went on to develop typical shoulder and hand symptoms, and a diagnosis of SHS was given. 5 weeks of physical therapy later, the patient had increased ROM in the shoulder and hand. Shoulder pain persisted but at a reduced level. | Limited follow-up information, 2/3 patients had improved but not resolved symptoms |
| Van der Korst, J | 1966 | 75 | Several | The duration of treatment (predominantly phenobarbital) and onset of SHS ranged from a few weeks to 20 years. Of those treated with phenobarbital, 5/25 had unilateral SHS, and all had resolved symptoms within 6 months. For the remaining 20 pts (all with bilateral SHS), the hypertrophic stage (i.e. typical hand/shoulder symptoms) persisted for 3-9 months, followed by atrophic changes to the hand, and digital contractures. | No follow-up reported |
| Mowat, A | 1974 | 3 | Several | **Case 1:** (Idiopathic) Patient developed shoulder symptoms without apparent cause, symptoms progressed to include hand over the next 7 months. Patient underwent several treatments, and all symptoms slowly improved over the next 12 months.  **Case 2:** (Stroke): Time between stroke and onset of symptoms is not reported; but patient reports severe shoulder and hand symptoms, including flexion deformity in the digits. With treatment, over the next 11 months their symptoms improved, but there was residual limited abduction in shoulder and flexion deformities, however, no pain in the shoulder or hand.  **Case 3:** (Epilepsy, physical trauma, surgery) Patient had an epileptic seizure in her sleep and significantly damaged her shoulder, woke up with limited ROM and pain in the shoulder. She had surgery 3 months later to repair damage; within 2 weeks she had developed SHS hand symptoms. Initial treatment was unsuccessful, and symptoms persisted for a further 3 months. New treatment was initiated and symptoms improved over next 9 months – no pain, but reduced ROM in shoulder and hand, accompanied by flexion contractures in the digits, but generally function was unimpaired. | No further follow-up after initial 11/12 month period |
| Davis, S | 1977 | 540 | Stroke | Of all 68 SHS patients, time from onset of stroke to first SHS symptoms: 1-2 months (28%), 2-3 months (37%), 3-4 months (16%), 4-5 months (17%) and 5-6 months (2%). Authors undertake a programme of oral steroids and intensive rehab. All 68 patients became pain free within three weeks. 60% of patients had some partial motor loss after treatment, but this is mainly attributed to their hemiplegia rather than SHS. Authors note that any symptom relapse in nine patients after treatment was addressed with a new course of treatment. | No follow-up reported |
| Low, L | 1978 | 1 | Sterilisation | After a laparoscopic sterilisation procedure, patient reported pain in the right shoulder. This progressed five weeks later to pain and swelling in the hand, with trophic symptoms, and limited ROM in both shoulder and hand.18 months after initial symptoms, and unsuccessful treatment, symptoms suddenly worsened with pronounced pain on moving hand or shoulder. Prednisolone was administered and symptoms improved. | Residual limited ROM in the shoulder and swelling in the hand persisted ~11 months later |
| Walker, J | 1983 | 3 | Cerebral neoplasms | **Case 1:** Patient had surgery to remove astrocytoma – five months later they reported shoulder pain and swelling in the hand. Six months later, symptoms progressed to limited ROM in the shoulder and flexion contractures in the hand. Course of prednisolone was started for 3 months then tapered off. Approximately 4 months later, patient had complete resolution of hand swelling and significantly reduced pain in the affected shoulder.  **Case 2:** Patient had surgery to remove a large melanoma – 2 months later they developed pain and limited ROM in the shoulder, and pain and swelling in the hand. A course of prednisolone was started for 2 weeks, wherein pain and motion were improved in the shoulder, but flexion contractures developed in the digits. After 3 months of prednisolone (dose was gradually reduced and stopped), shoulder symptoms now appeared in the left shoulder. Six months after prednisolone was restarted, shoulder pain was improved, and hand swelling was resolved. Limited ROM and flexion contractures in the hand/digits persisted.  **Case 3:** Patient had surgery to remove an astrocytoma – 10 months later, they reported typical shoulder and hand symptoms. Patient underwent a course of steroids for a month, and pain and ROM in the shoulder improved. However, their residual shoulder pain prevented them from engaging with physical therapy. Upon final examination [time unknown], patient had severe contractures in shoulder and hand that were irreversible. | No detailed information available for follow up – authors describe “final examinations,” but no timeframe is provided. All cases had residual shoulder/hand symptoms, one case had severe contractures |
| Taggart, A | 1984 | 2 | Ovarian carcinoma | **Case 1**: Approximately 10 months after a hysterectomy & bilateral salpingo-oophorectomy for ovarian cancer, patient reported pain and swelling in the hand, classic trophic changes, and limited ROM in the shoulder. Treatment was unsuccessful, and symptoms persisted until their death 10 months later.  **Case 2:** 3 weeks after surgery, patient reported swelling of hands and stiffness in the shoulders. Symptoms progressed over next 3 months, including trophic changes and flexion contractures in the hands. Treatment was unsuccessful, and 7 months later hands had formed severe flexion deformities which surgery could not fix. Symptoms persisted for the next 12 months until their death. | No follow-up available |
| McGill, P | 1985 | 1 | Ovarian carcinoma | Patient underwent a course of chemotherapy for ovarian cancer, and 5 months later reported pain in hands and shoulders; this progressed to severe swelling and flexion deformities in her hands. Prednisolone improved her shoulder discomfort, but had minimal effect on her hands, which remained the same until her death 3 months later. | No follow-up available |
| Reddy, M | 1985 | 1 | Phenobarbital | A patient with epilepsy was prescribed phenytoin and phenobarbital, 2 months after their phenobarbital dose was increased, they reported pain, swelling and stiffness in multiple joints. Physical therapy and pain management temporarily improved these symptoms. 2 months later this progressed to pain, swelling, and stiffness of shoulder and hands, accompanied by trophic changes and flexion contractures in the hand. Multiple treatments (including SGB) were applied, and pain and swelling were reduced within 6 weeks. At the end of her observation, she had only extremely mild limited ROM in her PIP joints of the left hand. | No detailed information available for follow up – authors describe a “final observation,” but no timeframe is provided. |
| Chalsen, G | 1987 | 41 | Stroke | Time interval between stroke to development of SHS, on average, was 8 weeks. Authors note that the SHS assessment was usually performed about 8 weeks after the initial stroke. | No follow-up reported |
| Aisen, P | 1994 | 43 | Spinal cord injury | Mean time between injury and admission to rehab unit was 60 days, the mean time from admission to onset of pain (shoulder and/or hand) was 24 days. 14/27 pts experienced onset of pain in their first week after admission. | No follow-up reported |
| Braus, D | 1994 | 132 | Stroke | In the majority of patients, shoulder pain developed in the first or second month after the stroke, and SHS developed between the second and third months after hemiplegia. | Majority of patients (31/34) treated with medication and therapy remained symptom free up to 6 months after discharge |
| Valley, M | 1994 | 1 | Brachial plexus injury | Patient sustained brachial plexus injury during surgery, reported severe pain in the shoulder and hand afterwards. Pain was resolved after a sympathectomy. 4 years later, the pain returned after minor trauma to the shoulder. Shoulder and hand symptoms returned, but more severe, including trophic/vasomotor changes in the hand. Several anaesthetic options were explored, including SGB, shoulder pain persisted at a baseline of 3/10, with 20% hyperalgesia/allodynia in the rest of the limb. [Exact timeline unknown] | No follow-up reported |
| Aisen, M | 1995 | 14 | Spinal cord injury | Average time from inciting injury to the onset of SHS was 54 days (7.7 weeks). Mean time from injury to author investigation was 3.6 months. | No follow-up reported |
| Hesse, S | 1995 | 39 | Stroke | Not reported | No follow-up reported |
| Dekker, J | 1997 | 9 | Stroke | Mean time between onset of stroke and onset of shoulder pain (in group that developed SHS) was 5.1 weeks | No follow-up reported |
| Desantis, A | 2000 | 234 | Phenobarbital | The time mean time between beginning phenobarbital treatment and developing SHS was 88.8 days. The mean duration of symptoms was 129 days – though this varied depending on the associated neurological condition, for example, for patients with aneurysms symptom duration was 213 days. | 5-year follow-up was reported, but no information was available on SHS symptoms |
| Kondo, I | 2001 | 152 | Stroke | 38/152 patients developed SHS within a period of 4 months from the onset of stroke. | 5-month follow-up was reported, but no information was available on SHS symptoms |
| Massarotti, M | 2008 | 1 | Gastric cancer | Patient presented with 2-month history of pain in shoulder, and pain and swelling in hand. Patient was diagnosed with cancer and underwent chemotherapy, and signs and symptoms of SHS were resolved 2 months later. SHS symptom duration was 4 months. | No follow-up reported |
| Santamato, A | 2009 | 1 | Stroke | 8 weeks after stroke, patient experienced pain, swelling and trophic/sudomotor changes in the hand, and a diagnosis of CRPS-SHS was given. After 6 months of treatment, signs of SHS improved; but pain and limited ROM in the digits remained. | No follow-up reported |
| Akasbi, N | 2010 | 1 | Lung cancer | Patient was in an accident resulting in several fractures in both arms and underwent surgery. Over the course of 10 days, they developed severe pain and limited motion in the hands and shoulders, with significant sweating and swelling in both hands. Further investigation revealed lung cancer. After receiving various treatments for SHS (lung cancer was terminal), 2 months later symptoms of SHS were resolved. | No follow-up reported |
| Hartwig, M | 2012 | 41 | Stroke | Within 49 days of having a stroke, 22/41 pts had developed shoulder hand syndrome (symptom duration - max recorded was 28 days) | No follow-up reported |
| Wang, J | 2015 | 120 | Stroke | Limited information provided - SHS symptoms developed in 120 patients within 6 months of a stroke | No follow-up reported |
| Pan, J | 2020 | 90 | Stroke | On average, pts developed SHS within 35 days of having a stroke. All groups showed an improvement of symptoms after 4 weeks of treatment, though some symptoms persisted. | No follow-up reported |

**Table A6**: Description of onset, duration, and resolution of shoulder-hand syndrome symptoms in all included articles

|  |
| --- |
